# ProBDNF as an Indicator of Improvement among Women with Depressive Episodes

**DOI:** 10.3390/metabo12040358

**Published:** 2022-04-16

**Authors:** Weronika Zwolińska, Maria Skibinska, Agnieszka Słopień, Monika Dmitrzak-Węglarz

**Affiliations:** 1 Department of Child and Adolescent Psychiatry, Karol Jonscher Clinical Hospital, Poznan University of Medical Sciences, Szpitalna 27/33 St., 60-572 Poznan, Poland; agaslopien@ump.edu.pl; 2 Department of Psychiatric Genetics, Medical Biology Center, Poznan University of Medical Sciences, Rokietnicka St. 8, 60-806 Poznan, Poland; mariaski@ump.edu.pl (M.S.); mweglarz@ump.edu.pl (M.D.-W.)

**Keywords:** mood disorders, depression, bipolar disorder, brain-derived neurotrophic factor, biomarkers

## Abstract

Depression is a chronic psychiatric disorder with a heavy socioeconomic burden. Studies on biomarkers are needed to comprehend the pathophysiology of depression and to improve treatment outcomes. Research points to the importance of imbalance between mature brain-derived neurotrophic factor (BDNF) and its precursor, pro–brain–derived neurotrophic factor (proBDNF), in the pathophysiology of mood disorders and the potential neurodegenerative role of calcium-binding protein B (S100B). Our objective was to compare BDNF, proBDNF, and S100B serum levels before and after the treatment of acute depressive episodes and to assess their correlation with the severity of symptoms and history of stress. We also aimed to investigate the differences in BDNF, proBDNF, and S100B levels between depression in the course of bipolar disorder (BD) and major depressive disorder (MDD). We recruited 31 female patients diagnosed with BD or MDD who were hospitalized due to current depressive episodes. The patients had their serum BDNF, proBDNF, and S100B levels evaluated using the ELISA method upon admission and after the symptoms improved, at discharge. We found that proBDNF levels decreased significantly with the treatment (*p* = 0.0478), while BDNF and S100B levels were not altered significantly. No differences in biochemical parameters between MDD and BD subjects were observed. Consequently, we concluded that a decrease in serum proBDNF levels could be considered a biomarker of recovery from depressive episodes.

## 1. Introduction

Affective disorders are chronic psychiatric conditions that exert a significant impact on health and the economy worldwide [1]. The general category of affective disorders is divided into depressive disorders, including major depressive disorder (MDD) and bipolar disorder (BD). Both MDD and BD may manifest with the recurrence of depressive episodes characterized mainly by depressed mood, anhedonia, and energy loss, while additional occurrences of at least one episode of elevated mood is diagnostic of BD [2]. The risk of suicide among patients suffering from depressive episodes is more than ten times higher than in the general population. Additionally, depression is associated with a severe increase in the rate of mortality from natural causes. The mortality data for BD are, in fact, comparable with that for heavy smoking [3]. Consequently, life expectancy is significantly reduced in mood disorders, making their effective treatment a fundamental goal for psychiatry. Studies on biomarkers of treatment response in mood disorders have yielded promising results, making the future of personalized treatment in psychiatry more feasible.

One of the most thoroughly investigated biomarkers in mood disorders is the brain-derived neurotrophic factor (BDNF). BDNF is a protein from the neurotrophin family that plays an essential role in neurodevelopmental and neuroplastic processes. BDNF is first synthesized as a precursor protein pro-brain-derived neurotrophic factor (proBDNF) that is further processed into the mature form by proteases. BDNF stimulates neuronal adaptation, new cell formation, and elimination of unnecessary neurons, while proBDNF induces neuronal death and synaptical pruning. BDNF and proBDNF elicit their opposing effects via the tropomyosin-related kinase B receptor (TrkB) and neurotrophin receptor p75 (p75NTR), respectively [4]. Studies consequently report decreased BDNF levels among subjects suffering from MDD, which can be restored with successful antidepressant treatment [5,6]. Regarding bipolar depression, a large meta-analytical study showed that compared to healthy controls, peripheral BDNF level is reduced in manic and depressive episodes, while it is not significantly altered in euthymia [7]. These notions suggest the possibility of using serum BDNF levels as an indicator of disease activity and treatment response. However, studies show that the changes in BDNF following successful treatment are more evident in MDD than in bipolar depression [6]. More studies are required to verify whether the changes in BDNF could be used as indicators of treatment response in BD depression.

More recently, many studies have focused on the role of proBDNF in the pathophysiology and treatment of mood disorders [8]. For instance, the research on rodent models suggests that antidepressants might exert their therapeutic effect by restoring the brain’s balance between BDNF and proBDNF [9]. Moreover, a late study performed by Gelle et al. proved that BDNF and proBDNF serum levels vary inversely during antidepressant treatment in MDD [10]. There has been no study investigating the changes in proBDNF following the treatment of depressive episodes in BD.

Another promising biomarker in mood disorders is calcium-binding protein B (S100B), a protein predominantly produced and secreted by astrocytes in the central nervous system. It acts as a stimulator of cell proliferation and migration and as an inhibitor of apoptosis and differentiation. It is considered to have significant implications during brain development and regeneration of brain damage [11]. Due to these characteristics, S100B can be regarded as a marker for glial alterations proposed as a pathophysiological mechanism responsible for the emergence of mood disorders [12]. In support of this hypothesis, studies consistently show that S100B is elevated in mood disorders, more evidently in MDD than in BD [13,14,15]. Nevertheless, S100B was also found to be higher in BD patients during manic/depressive episodes when compared to euthymic BD patients [16]. Studies have also investigated the relationship between S100B changes and treatment outcome and found that higher baseline S100B levels indicated a better response to antidepressant treatment in MDD [17,18]. Although research on rodent models has shown that antidepressants might reverse an increased level of S100B [19], studies on human subjects with depression were not as coherent [17,18]. On the other hand, Tsai and Huang (2017) found that S100B levels decreased while treating bipolar patients presenting manic episodes [20]. More studies are needed to verify the dynamics of S100B serum changes among patients with depression.

BD may be frequently misdiagnosed as MDD, especially when a patient suffers from recurrent depression and is premorbid of a manic episode. Misdiagnosing BD as MDD could often be one of the reasons for resistance to treatment with selective serotonin reuptake inhibitors (SSRIs). Although both disorders are clinically related, the treatment differs, with mood stabilizers being the principal treatment for BD and SSRIs for MDD. Therefore, it comes as no surprise that studies have focused on searching for specific indicators that could aid in distinguishing between MDD and BD and could reduce the rates of misdiagnosis between these two conditions. As mentioned above, studies performed on the MDD population have consistently demonstrated that BDNF circulating levels are significantly lower in blood samples of MDD patients than in the healthy control group [21]. Since this relation is not so evident in BD patients, some authors suggest that BDNF levels might be a tool used to distinguish depression in the course of BD from MDD [22]. However, on the meta-analytic level, there was no difference in BDNF levels between the acute mood episodes in the course of BD and MDD [6].

The primary objective of this prospective study was to verify whether the serum levels of S100B, BDNF, and proBDNF change with the successful treatment of depressive episodes among women with BD and MDD. We also aimed to verify whether S100B, BDNF, and proBNDF levels correlate with the severity of depressive symptoms and the history of stress. Secondly, we also researched the differences in BDNF, proBDNF, and S100B levels between BD and MDD patients with depressive episodes.

## 2. Results

### 2.1. Characteristics of the Studied Group

A total of 31 subjects were included in the study. All of them presented with depressive episodes: 15 in MDD and 16 in the course of BD (Table 1). All included patients were females. There were no statistical differences in the MDD vs. BD groups regarding age, BMI, history of hospitalizations, comorbidities, marital history, education levels, or smoking history (Table 1). The stage of the disease varied across the patients, with the number of hospitalizations in the patients’ history extending from 1 to 46. Of the patients, 30 out of 31 were assessed with HDRS17 at baseline and after symptom improvement and 26 out of 31 patients fulfilled the BDI questionnaire. The mean time necessary to achieve remission in the two groups of patients is presented in Table 1. The median results and ranges of Hamilton Depression Rating Scale (HDRS), Beck Depression Inventory (BDI) and Brief Life Events Questionnaire (BLEQ) in the two studied groups are presented in Table 1. HDRS and BDI scores were significantly lower during the control visits for both groups of patients. In the study, 17 out of 31 patients filled in the BLEQ, and the scores varied from 2 to 12 with a median of 7. The medication status of the studied subjects is presented in Table 2. The patients were heterogenic in terms of medications and treated with different types of antidepressants, anticonvulsants, antipsychotics, or lithium carbonate. We lacked information about the initial medication status in the case of 11 out of 31 patients due to two main reasons: first, some patients had not been able to determine their current medication status upon admission and they did not provide any medical documentation; second, some patients had stopped their treatment before admission to a hospital due to non-compliance and there had been a significant wash-out period.

### 2.2. Biochemical Parameters

**Figure 1:**
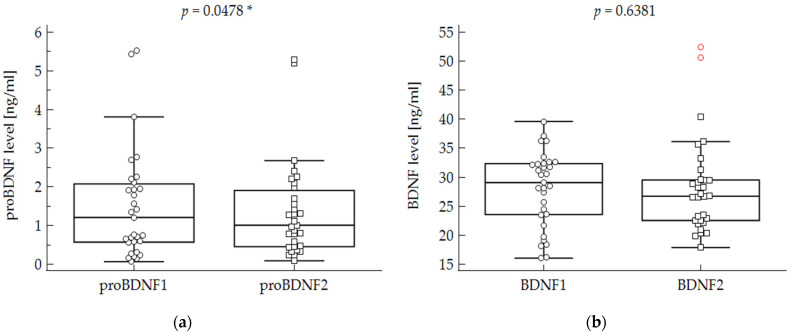
(**a**) Pro-brain-derived neurotrophic factor serum levels before (proBDNF1) and after (proBDNF2) the treatment of depressive episodes in the studied group overall. (**b**) Brain-derived neurotrophic factor serum levels before (BDNF1) and after (BDNF2) the treatment of depressive episodes in the studied group overall. Each value is presented as a circle in the first assessment and as a square in the second assessment. *—statistically significant result.

The biochemical assessments in acute depression (t1) and after improvement at discharge (t2) are presented in Table 3. The BDNF and S100B levels did not significantly differ after symptom improvement in the studied group. There were no statistical differences in the BDNF and S100B levels in the group overall, or after stratifying patients regarding the disease type (Table 3). The ProBDNF level was significantly lower during the second assessment when the studied group was analyzed overall but not in the subgroups of BD and MDD patients (Figure 1). There were no statistical differences in proBDNF, BDNF, and S100B levels between the patients with MDD and BD (Table 1).

### 2.3. Correlation with Stress and Depressive Symptoms

**Figure 2:**
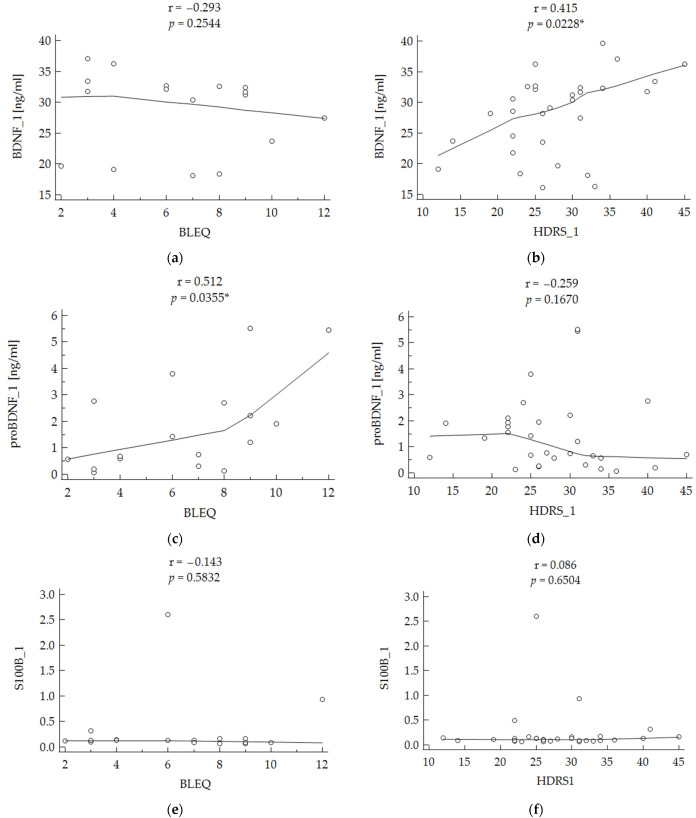
Correlation of biochemical parameters with stress and depressive symptoms overall. HDRS 1—Hamilton Depression Rating Scale before the treatment; BLEQ—Brief Life Event Questionnaire. BDNF—brain-derived neurotrophic factor, proBDNF—pro-brain-derived neurotrophic factor, S100B—calcium-binding protein B. First evaluation of BDNF, proBDNF, and S100B was used in the analysis. The Spearman Rank–Order Correlation Test was performed for all analysis. Statistically significant results were marked with *. (**a**,**c**,**e**)—correlation between BDNF, proBDNF, S100B respectively and the level of stress measured with BLEQ. (**b**,**d**,**f**)—correlation between BDNF, proBDNF, S100B respectively and the level of depressive symptoms measured with HDRS.

The correlation analysis revealed that the level of BDNF at baseline correlated positively with the HDRS depression symptom score (r = 0.415; *p* = 0.0228) (Figure 2b). There was no correlation between other biochemical parameters and the level of depression symptoms. There was, however, a positive correlation between the level of proBDNF and BLEQ score (r = 0.512; *p* = 0.0355), and it was the only parameter that significantly correlated with the history of stressful life events (Figure 2c).

## 3. Discussion

Our study found proBDNF levels to decrease with successful treatment of depressive episodes. Therefore, our results might suggest that proBDNF could be considered an important factor in the process of recovery from depressive episodes and serve as an indicator of improvement. As expected, we observed that proBDNF decreases with the treatment, which might have a beneficial effect on patients, given that proBDNF has been shown to induce neurodegenerative processes [23]. Our results align with previous studies reporting a decrease in proBDNF levels after treatment of depressive episodes [10]. Consistently, Jiang et al. reported a decrease in serum proBDNF level after eight weeks of antidepressant treatment among MDD patients [24]. On the other hand, a study performed by Yoshimura et al. failed to prove any changes in proBDNF following the period of antidepressant treatment of drug-naïve MDD patients [25].

To the authors’ knowledge, no study has investigated the dynamics of proBDNF changes following the treatment of bipolar depression specifically. Our study did not prove any differences in the proBDNF levels after stratifying the patients regarding the type of disease (BD/MDD). This might be due to the smaller sample size and consequently lower statistical tests’ power. More studies on larger groups are essential to address the question of whether proBDNF level changes differently among MDD and BD patients.

Interestingly, we also found proBDNF to correlate positively with the history of stress among the study subjects. Stress is an environmental factor well-known for its notorious part in the development of psychiatric disorders, possibly through altering the expression of stress-related genes, such as *BDNF* [4,26]. The neurotrophic theory of depression initially assumed that environmental stress decreases BDNF levels in the brain, which results in decreased neuroplasticity and morphological changes, such as hippocampal shrinkage [27]. Recently, it has been suggested that depression is not only caused by decreased BDNF levels but also by an increase in its precursor proBDNF [8,28]. Even though the positive correlation between proBDNF and the history of stress matches this hypothesis, our results should be interpreted with caution and considered more as a possible trend that requires further investigation, owing to the small sample of patients that were involved in this analysis (only 17 patients were included while the minimum sample size necessary to achieve adequate statistical power was calculated for 27).

Studies have previously shown the potential of using BDNF alone as a marker of depressive symptoms in MDD as well as in BD, although with mixed results [6,7]. The heterogeneity between the studies might have been caused by using BDNF ELISA kits unable to distinguish between proBDNF and its mature form. Consequently, earlier studies operated on combined levels of proBDNF and mature BDNF [22,29]. In one of the first studies, Zhou et al. reported that upregulation of proBDNF might be typical for MDD along with downregulation of BDNF [8]. Interestingly, in the study on two large independent cohorts of BD patients, Södersten et al. revealed the opposite [22]. Therefore, a major role of BDNF and proBDNF in differentiating BD from MDD has been proposed [30]. Our study failed to prove any differences in BDNF and proBDNF levels between MDD and BD patients. However, the analysis was significantly underpowered; therefore, we might have obtained falsely negative results. More studies on larger groups of patients are needed to address the question of differences in BDNF and proBDNF levels between MDD and BD patients. It is recommended that future studies separate BDNF from proBDNF, given their opposing effects, and the potential role of their interaction in the pathogenesis of BD and MDD described in the literature.

Our study did not demonstrate any differences in the S100B level before and after the treatment of an acute depressive episode. To the best of our knowledge, no other study investigated the dynamics of S100B changes during the treatment of acute depressive episodes in the course of BD. Nonetheless, it has been demonstrated that the S100B level could successfully differentiate BD patients in the acute manic or depressive state from euthymia [16]. Therefore, we assumed that the S100B level would significantly change with successful treatment, as shown in the study on patients with manic episodes [20]. Although we failed to confirm this hypothesis, our results are coherent with the study on the treatment of depressive episodes in MDD, where the authors proved no significant differences in the S100B level before and after the successful antidepressant treatment [18]. However, we must acknowledge the possibility of a second type of error in our analysis since the statistical power of applied tests was insufficient due to the small sample size. More studies are needed to answer the question of whether the S100B level changes significantly with the treatment of depressive episode and differs among BD and MDD patients.

The main limitation of our study was a small group of subjects, heterogeneous in terms of treatment. Even though our study included women only, it may be considered an advantage, given the different courses of the disease between genders [31]. Furthermore, we lacked a control group of healthy individuals.

## 4. Materials and Methods

### 4.1. Population

The studied group was recruited in 2017–2019 at the Department of Adult Psychiatry of Poznan University of Medical Sciences. All the participants were of Caucasian origin. The study was performed following the ethical standards established in the Declaration of Helsinki and was reviewed and approved by the Bioethics Committee of the Poznan University of Medical Sciences (resolution no. 758/17). All participants gave written informed consent before participating in the study. Participants were recruited from patients hospitalized with depressive episodes in the course of F.31 (bipolar disorder) or F.33 (recurrent depressive disorders) according to International Classification of Diseases (ICD-10). A consensus lifetime diagnosis was made by two psychiatrists according to the ICD-10 and Diagnostic and Statistical Manual of Mental Disorders (DSM-IV) criteria, using SCID (Structured Clinical Interview for DSM Disorders) [32] and OPCRIT (the Operational Criteria Diagnostic Checklist) [33]. The Hamilton Depression Rating Scale (HDRS) and/or Beck Depression Inventory (BDI) were used to assess the severity of depression symptoms. All participants were evaluated on admission (t1) and after clinical improvement at discharge (t2). An HDRS scoring <8 was required for achieving clinical remission or at least 50% score reduction, defined as treatment response [34]. The history of stressful life events was assessed using the Brief Life Events Questionnaire (BLEQ) [35]. Demographic data on age, gender, education, marriage, and family history of psychiatric disorders were collected (Table 1). The patients were treated according to their physicians’ decisions, and participation in the study did not influence the treatment choices. Patients enrolled in the study also had to meet the following inclusion/exclusion criteria: ages 18–65 years, at least the second episode in lifetime history, and no chronic or acute somatic or neurological diseases.

### 4.2. Biochemical Analysis

Biochemical analysis was performed upon admission and after the improvement in their symptoms. All patients had their sera analyzed for BDNF, proBDNF, and S100B levels. Ten milliliters of peripheral venous blood of each fasting participant was collected into anticoagulant-free tubes between 7 a.m. and 9 a.m. After 1 h incubation, serum was separated by centrifugation, aliquoted, and stored at −70 °C until further analyses. Enzyme-linked immunosorbent assays were performed using BDNF_DuoSet human/mouse (cat. no DY248), proBDNF_DuoSet human (cat. no DY3175), S100B_DuoSet human (cat. no DY1820-05), and ELISA Development Kit (R&D System, Minneapolis, MN, USA), according to manufacturer’s instructions. Plates were blocked for 3 h in reagent diluent (1% bovine serum albumin (BSA)/phosphate buffered saline (PBS)) and incubated overnight with 100 µL of samples at 4 °C with shaking. Samples were diluted 1:120 for BDNF and 1:2 for proBDNF and S100B to fit the standard curve range. All plates were run within one week on the same kit lot by the same experienced operator. Standard curves for all analytes ranged from 1000 to 15.6 pg/mL. Intra-assay and inter-assay variability were <5% coefficient of variation (CV) and <10% CV, respectively.

### 4.3. Statistical Analysis

Statistical calculations were made using the MedCalc^®^ Statistical Software version 19.5.6 (MedCalc Software Ltd., Ostend, Belgium). The distribution of variables was studied by the Shapiro–Wilk test. Non-parametric Wilcoxon signed-rank test for two dependent groups was performed for all variables that did not meet the normal distribution criteria for statistical comparisons. A comparison of two unpaired groups was performed using the Student’s T-test (for the data that followed normal distribution) or U Mann–Whitney test (for non-parametric variables). Nominal data were analyzed using the χ^2^ test. Spearman’s rank correlation coefficient was applied to assess the relationship between the analyzed variables. The significance level was set at α < 0.05 for all analyses.

## 5. Conclusions

A decrease in serum proBDNF level could be considered a biomarker of recovery from depressive episodes.

## Figures and Tables

**Table 1:** Characteristics of the studied groups.

	Bipolar Disorder (BD)	Major Depressive Disorder (MDD)	*p*-Value (BD vs. MDD)
Number (*n*)	16	15	
Sex	females	females	
Age (years)	34.44 ± 14.27	44.67 ± 17.36	0.0749
BMI (kg/m^2^)	22.78 ± 3.83	25.39 ± 5.10	0.1063
Tobacco smokers (yes/no/no data) (*n*)	4/10/2	5/10/0	0.7855
Educational level (*n*) None or low	2	3	0.8319
Middle	10	8	
High	4	4	
Marital status (*n*)			0.2828
single	9	5	
married	4	8	
divorced	2	1	
widowed	0	1	
no data	1	0	
Somatic disease (yes/no)	9/7	9/6	0.4719
Family history of psychiatric illness (yes/no)	12/4	10/5	0.6153
History of hospitalizations (*n*)			0.5710
1–4	12	14	
5–10	1	0	
>10	1	1	
Time to achieve remission (days)	52 (±30)	61 (±73)	0.6783
BDI (t1/t2) *	31.5 (19–44)/6 (0–29)	28 (13–53)/10.5 (3–26)	0.2848/0.0506
HDRS (t1/t2) *	30 (12–45)/3 (0–6)	26 (14–36)/3 (1–19)	0.4541/0.3661
BLEQ *	7 (2–9)	7 (3–12)	0.3323
proBDNF **	1.56 (±1.49)	1.46 (±1.38)	0.8744
BDNF **	30.04 (±5.99)	26.12 (±6.46)	0.0913
S100B **	0.33 (±0.66)	0.15 (±0.11)	0.9525

*n*—number, t1—on admission, t2—at discharge, BMI—Body Mass Index, BDI—Beck Depression Inventory, HDRS—Hamilton Depression Rating Scale, BLEQ—Brief Life Event Questionnaire, proBDNF—pro-brain-derived neurotrophic factor, BDNF—brain-derived neurotrophic factor, S100B—calcium-binding protein B, *—data expressed as median and minimum–maximum range, **—data expressed as an average and standard deviation score.

**Table 2:** Medication status on admission (t1) and follow-up (t2).

Medication Status of the Studied Group	T1/T2
none	1/0
AD	6/4
AP	2/0
AD + AP	5/8
AD + Li	1/1
AD + AC	1/2
AP + AC	0/1
AD + AP + Li	1/4
AD + AC + Li	2/2
AD + AP + AC	1/6
AD + AP + AC + Li	0/1
No data	11/2

AD—antidepressant, AP—antipsychotic, AC—anticonvulsant, Li—Lithium carbonate.

**Table 3:** Biochemical parameters in acute depressive episode before treatment (t1) and after the improvement of symptoms (t2).

Measure (ng/mL)	MDD		*p*-Value T1/T2	BD		*p*-Value T1/T2	Patients Overall		*p*-Value T1/T2
	T-1	T-2		T-1	T-2		T-1	T-2	
BDNF	30.04 (±5.99)	29.57 (±8.21)	0.3303	26.12 (±6.46)	26.94 (±8.09)	0.7436	28.02 (±6.45)	28.21 (±8.12)	0.6381
proBDNF	1.56 (±1.49)	1.39 (±1.26)	0.1205	1.46 (±1.38)	1.36 (±1.30)	0.2522	1.51 (±1.41)	1.37 (±1.26)	**0.0478**
S100B	0.33 (±0.66)	0.32 (±0.62)	0.6221	0.15 (±0.11)	0.15 (±0.10)	0.3910	0.24 (±0.48)	0.23 (±0.44)	0.7800

Data expressed as mean and standard deviation. Analysis was made using the Wilcoxon signed-rank test. Statistically significant results are marked in bold. T-1—depressive episode before treatment, T-2—after the improvement of symptoms, T1/T2—the difference between T-1 and T-2, BDNF—brain-derived neurotrophic factor, proBDNF—pro-brain-derived neurotrophic factor, S100B—calcium-binding protein B.

## Data Availability

The data presented in this study are available from the corresponding author upon request due to the ongoing project and continuation of the studies.
